# ePHex: a phase 3, double-blind, placebo-controlled, randomized study to evaluate long-term efficacy and safety of *Oxalobacter formigenes* in patients with primary hyperoxaluria

**DOI:** 10.1007/s00467-022-05591-5

**Published:** 2022-05-12

**Authors:** Gema Ariceta, Laure Collard, Saoussen Abroug, Shabbir H. Moochhala, Edward Gould, Abir Boussetta, Mohamed Ben Hmida, Sudarsana De, Tracy E. Hunley, Faical Jarraya, Gloria Fraga, Ana Banos, Elisabeth Lindner, Bastian Dehmel, Gesa Schalk

**Affiliations:** 1 Hospital Vall d’Hebron, Barcelona, Spain; 2 Centre Hospitalier Umniversitaire de Liege, Liege, Belgium; 3 Hôpital Universitaire Sahloul, Sousse, Tunisia; 4 Royal Free Hospital, London, UK; 5 Vanderbilt University Hospital, Nashville, USA; 6 Charles Nicolle University Hospital, Tunis, Tunisia; 7 Hedi Chaker University Hospital, Sfax, Tunisia; 8 Nottingham Children’s Hospital, Nottingham, UK; 9 Hospital Sant Pau, Barcelona, Spain; 10 OxThera Intellectual Property AB, Stockholm, Sweden; 11 Kindernierenzentrum, Bonn, Germany

**Keywords:** Primary hyperoxaluria, Oxalate, Oxabact, *Oxalobacter formigenes*, eGFR

## Abstract

**Background:**

Primary hyperoxalurias (PHs) are rare genetic diseases that increase the endogenous level of oxalate, a waste metabolite excreted predominantly by the kidneys and also the gut. Treatments aim to improve oxalate excretion, or reduce oxalate generation, to prevent kidney function deterioration. *Oxalobacter formigenes* is an oxalate metabolizing bacterium. This Phase III, double-blind, placebo-controlled randomized trial investigated the effectiveness of orally administered Oxabact™, a lyophilized *O. formigenes* formulation*,* at reducing plasma oxalate levels in patients suffering from PH.

**Methods:**

Subjects (≥ 2 years of age) with a diagnosis of PH and maintained but suboptimal kidney function (mean estimated glomerular filtration rate at baseline < 90 mL/min/1.73 m^2^) were eligible to participate. Subjects were randomized to receive Oxabact or placebo twice daily for 52 weeks. Change from baseline in plasma oxalate concentration at Week 52 was the primary study endpoint.

**Results:**

Forty-three subjects were screened, 25 were recruited and one was discontinued. At Week 52, *O. formigenes* was established in the gut of subjects receiving Oxabact. Despite decreasing plasma oxalate level in subjects treated with Oxabact, and stable/increased levels with placebo, there was no significant difference between groups in the primary outcome (Least Squares mean estimate of treatment difference was − 3.80 μmol/L; 95% CI: − 7.83, 0.23; *p*-value = 0.064). Kidney function remained stable in both treatments.

**Conclusions:**

Oxabact treatment may have stabilized/reduced plasma oxalate versus a rise with placebo, but the difference over 12 months was not statistically significant (*p* = 0.06). A subtle effect observed with Oxabact suggests that *O. formigenes* may aid in preventing kidney stones.

**Graphical abstract:**

A higher resolution version of the Graphical abstract is available as Supplementary information.

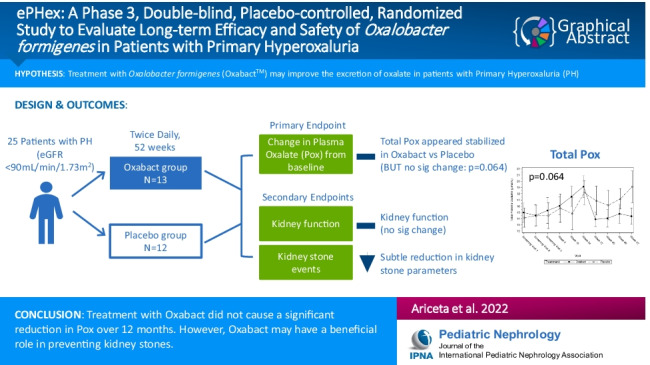

**Supplementary Information:**

The online version contains supplementary material including a graphical abstract available at 10.1007/s00467-022-05591-5.

## Introduction

The primary hyperoxalurias (PH) are rare metabolic, orphan diseases affecting 3 per million individuals in Western countries, with approximately 2500 to 5000 prevalent cases in the USA and Europe [1]. The three types of PH (types 1, 2, and 3) differ in severity and are caused by mutations in distinct genes encoding hepatic enzymes [2–4]. Loss of enzymatic function results in the overproduction of oxalate, a metabolic waste product that cannot be metabolized by the liver and is excreted predominantly via the kidneys.

Increased oxalate concentration results in the formation of calcium oxalate (CaOx) crystals, which accumulate within the kidney parenchymal tissue causing recurrent nephrolithiasis and/or progressive nephrocalcinosis [5]. Tubular internalization of oxalate also induces inflammation, fibrosis, kidney function decline, and kidney failure [6, 7]. Estimated glomerular filtration rate (eGFR), a measure of kidney function, inversely correlates with plasma oxalate (Pox) concentration in patients with early-stage chronic kidney disease (CKD; stages 1–3b), occurring before substantial loss of kidney function [8, 9]. Increased Pox concentration, due to impaired kidney function and reduced urinary oxalate (Uox) excretion, results in systemic CaOx deposition, which can cause osteopathy [2], retinopathy, cardiac oxalosis [10], and vasculopathy [11].

Treatments such as hyper-hydration and conservative medication (e.g., citrate, magnesium and, in a subset of patients, vitamin B6 [pyridoxine]) [2] aim to minimize systemic deposition of CaOx, but are limited in their ability to prevent kidney failure. Even in patients receiving chronic kidney replacement therapy, elimination of oxalate fails to counteract its continuous overproduction, ultimately resulting in systemic deposition of CaOx [12]. In PH patients with maintained kidney function, treatment strategies to prevent the development of kidney stones and kidney damage aim to reduce CaOx crystal formation. A recent treatment approach (lumasiran) involves RNA interference technology to reduce the production of glycolate, the main oxalate precursor in PH type 1 [13].

Although renal clearance is the primary means of oxalate elimination, excretion also occurs via the gastrointestinal (GI) tract [14]. *Oxalobacter formigenes* is a Gram-negative, non-pathogenic, anaerobic bacterium that forms part of the normal human intestinal flora, requiring oxalate as a substrate for its survival and growth. It promotes the secretion of oxalate from plasma into the intestine via the solute carrier anion transporter, SLC26, and facilitates the removal of endogenously produced oxalate from the blood [15–17].

Oxabact™ (OC5; OxThera Intellectual Property AB, Sweden) is an oral formulation of lyophilized *O. formigenes* genotype 1 (strain HC-1). Oxabact may enhance intestinal excretion of oxalate, potentially mobilizing stored oxalate and decreasing the total body burden and serum concentration of oxalate. Previous PH clinical studies have demonstrated the safety and tolerance of *O. formigenes* formulations and its ability to occupy the GI tract. Definitive evidence on its ability to reduce Uox excretion is lacking [18, 19]. However, post-hoc analysis has shown that treatment with *O. formigenes* stopped or delayed the natural rise of Pox in patients with CKD stage 2–4, and reduced Pox in CKD stage 5 patients [12]. Preventing the elevation of Pox levels in early-stage PH patients may stop or delay further deterioration of kidney function [20].

Here, we report 52-week treatment results from the OC5-DB-02 study, a Phase III, randomized, double-blind study evaluating the efficacy and safety of Oxabact versus placebo in stabilizing or reducing Pox concentration, stabilizing or improving kidney function and reducing oxalate deposits in subjects with PH and maintained kidney function below the lower limit of the normal range (eGFR < 90 mL/min/1.73 m^2^).

## Methods

### Subjects

This was a Phase III, double-blind, placebo-controlled, randomized, multi-center study. The study was initiated at eight European sites (Liège, Belgium; Lyon, France; Paris, France; Bonn, Germany; Amsterdam, Netherlands; Barcelona, Spain; Nottingham, UK; London, UK), three USA sites (Boston, MA, USA; Nashville, TN, USA; Rochester, MN, USA) and three African sites (Sfax, Tunisia; Tunis, Tunisia; Sousse, Tunisia). Study enrollment was between April 2018 and May 2021. The study was approved by regional regulatory authorities and local ethics committees and performed in accordance with the Declaration of Helsinki and the International Council for Harmonization of Technical Requirements for Pharmaceuticals for Human Use Good Clinical Practice guidelines.

Subjects ≥ 2 years of age with a diagnosis of PH, maintained kidney function below the lower limit of the normal range (eGFR < 90 ml/min/1.73 m^2^) but without risk for dialysis and a total Pox concentration ≥ 10 µmol/L were eligible to participate. The eGFR was calculated in children (< 18 years) using the Schwartz formula [21] and for adults (age ≥ 18 years) with the Chronic Kidney Disease Epidemiology Collaboration formula [22]. Subjects were not allowed to change vitamin B6 supplementation regimen. Those already receiving vitamin B6 had to be on a stable dose ≥ 3 months before screening and remain on that dose throughout study participation. Exclusion criteria included an inability to swallow the medication, transplant recipients (solid organ or bone marrow), subjects on, or close to dialysis, or subjects expected to require dialysis during the study. Subjects were also excluded if they required ongoing administration of antibiotics to which *O. formigenes* is sensitive, ascorbic acid preparations, or had a diagnosis of enteric (secondary) hyperoxaluria.

Pregnant women, women of childbearing potential not using adequate contraceptive precaution, subjects with a medical condition rendering them susceptible to adverse effects of study treatment or unable to follow study procedures, and subjects with a condition that was likely to interfere with the study treatment mechanism of action (such as abnormal GI function) were also ineligible for study participation.

### Study design

Patients with high Uox levels tend to have a worse disease progression rate [23]. Therefore, subjects were stratified by PH type (1, 2 or 3) and baseline Uox excretion of > or ≤ 1.87 mmol/1.73 m^2^ per day and were monitored during an 8-week baseline period prior to the initiation of study treatment. Information on pre-study kidney stone event–related symptoms was collected at the start of the study. Following screening and baseline evaluations, eligible subjects were randomized 1:1 to receive either Oxabact or placebo twice daily for 52 weeks. Blood samples for analyses of safety laboratory tests, Pox and kidney function were obtained at the study sites at screening/baseline and at each study visit scheduled every 8 weeks, up to Week 52. Subject quality of life (QoL) was evaluated at baseline and four times during the study (Weeks 8, 24, 40, and 52) using a 36-item short form survey (SF-36v2) in subjects ≥ 18 years old and using a 50-question child health questionnaire for parents (CHQ/PF50) of subjects < 18 years old. Traditional echocardiography (TE) and speckle tracking echocardiography (STE) were performed at screening/baseline, Week 24 and Week 48. Subjects provided stool samples for determination of *O. formigenes* cell counts, as well as 24-h urine samples at baseline and quarterly (Week 8, 24, 40, and 52) throughout the study. Kidney ultrasound was performed at screening/baseline and at Week 48.

### Treatment administration

All subjects in the study received Oxabact (active treatment) supplied as size 4 enteric-coated capsules or placebo capsules with the same appearance. One capsule, swallowed with water, was taken twice daily with food (breakfast and dinner). Each active treatment capsule contained lyophilized *O.* *formigenes*, strain HC-1 (≥ 10^9^ to < 5^10^ colony forming units per dose).

### Safety evaluation

Safety evaluation included physical examination, vital signs, and safety laboratory tests at baseline performed at every study visit. Adverse events (AEs) and prior/concomitant medications were monitored throughout the study. A Data and Safety Monitoring Board (DSMB) periodically reviewed blinded safety data, evaluated excess AEs, and assessed the overall integrity and conduct of the study.

### Study endpoints

The primary endpoint was the change from baseline (CfB) in total Pox concentration after 52 weeks of treatment. Secondary efficacy endpoints were the CfB in kidney function as determined by eGFR, and the change in frequency of kidney stone events after 52 weeks. Other endpoints included change in fecal *O. formigenes* cell number, myocardial function (assessed by echocardiography), free Pox concentration, nephrocalcinosis, and Uox after 52 weeks of treatment.

### Efficacy analysis

Total and ‘free’ plasma Pox was quantified by gas chromatography with mass selective detection, performed at the Academic Medical Center, University of Amsterdam, Netherlands. For total Pox, blood samples were acidified at site with concentrated HCl, within 1 h of collection, to solubilize oxalate, dissolve CaOx crystals and to prevent oxalate neogenesis under storage conditions at –80 °C. For free Pox, plasma samples were ultrafiltered at 10 kD, then acidified to prevent oxalate neogenesis and stored at − 20 °C.

At the central lab, Pox was extracted with ethylacetate before quantification. For free Pox, normal values are 3–5 µmol/L and for total Pox, normal values are 7–10 µmol/L. Additionally, post-hoc efficacy analysis of the total:free Pox ratio was also performed to determine changes in Pox deposits.

Genotyping (genotype I or II), and quantification of *O. formigenes* cell numbers were performed at the Institut for Microecology, Germany, using a previously described validated real-time quantitative polymerase chain reaction (qPCR) assay [24]. All other plasma analyses were performed at the Cerba Research, Gent, Belgium and urinary samples were analyzed at The Doctors Laboratory, London, UK.

### Kidney ultrasound analysis

Ultrasound of the kidneys was performed at the site hospital by BioClinica Inc., USA, validated operators. Images were evaluated by the contracted central imaging vendor (Bioclinica Inc.) to determine the grade of nephrocalcinosis and the number of kidney stones. For nephrocalcinosis, the grading system for determination by ultrasound used 0–3 grades (Grade 0 = no echogenicity, Grade 1 = mild echogenicity around medullary pyramid borders, Grade 2 = moderate echogenicity around and inside pyramids, Grade 3 = severe echogenicity of entire pyramids [25]). For kidney stones, nephrolithiasis was defined as the presence of calcifications located within the collecting system.

### Sample size

Based on previous studies [8, 12] and using a mixed-effect model of repeated measures, we estimated a requirement for 18 subjects allowing for a 5% significance level with 90% power. In total, 25 subjects were recruited, allowing for possible dropouts.

### Statistical methods

Primary endpoint analyses were performed using the full analysis set (FAS). Primary statistical analysis was performed using a Mixed model repeated measures (MMRM) model that included the following fixed effects: treatment group, baseline Pox value, stratification, week, and week-by-treatment interaction. The primary comparison was the CfB between Oxabact and placebo at Week 52. In addition, a repeated measures model was applied to determine the slopes through time for each of the treatments for the CfB value. For this model, the time factor was considered to be continuous and was expressed in years. A change in slope between the two treatments was assessed. A pre-defined first-order autoregressive (AR(1)) covariance structure was used for all models.

Mean (± standard error [SE]) total plasma values over time for both treatment arms were plotted in addition to individual concentration–time plots. Additionally, a generalized linear model analysis of covariance (ANCOVA) was done using the FAS as supportive analysis for the Week 52 CfB comparison. A sensitivity analysis for the primary endpoint at 52 weeks was performed using multiple imputation (MI) under the assumption of missing data being missing at random (MAR) prior to applying the MRMM analysis.

All secondary endpoint analyses were performed using the FAS. For the endpoints CfB in kidney function, CfB in free Pox and CfB in Uox excretion, analysis was performed by applying the same primary MMRM model as for the primary endpoint. For the endpoint ‘frequency of kidney stone events’, incidence rate difference (IRD) and incidence rate ratio (IRR) were calculated, and a zero-inflated Poisson model was used for comparison between treatment arms on the total number of kidney stone events. Analysis for the percent CfB comparison between treatment arms for total Pox was done using an ANCOVA. Comparison between the treatment arms for the ‘subjects achieving near-normalization of total Pox concentration’ was performed by using the stratified generalized Cochran–Mantel–Haenszel test of general association. Descriptive statistics were presented for all endpoints. Correlation analyses between eGFR and total Pox concentration, left ventricular ejection fraction (LVEF) and total Pox concentration, and global longitudinal strain (GLS) and total Pox concentration were performed.

## Results

### Study population

**Fig. 1:**
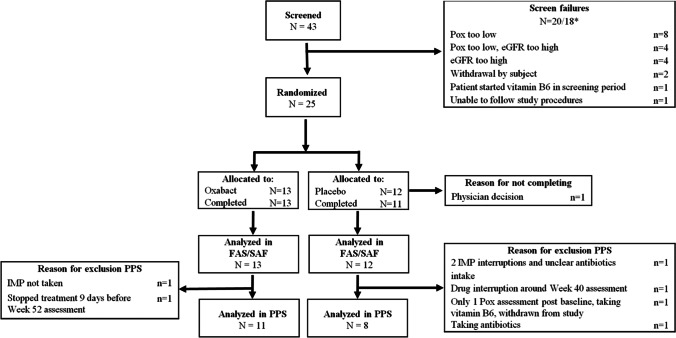
CONSORT diagram showing subject disposition. *There were two initial screen failures (one subject had an initial eGFR too high and one subject had an initial Pox that was too low); the subjects were rescreened and different subject numbers were obtained. eGFR = estimated glomerular filtration rate; FAS = full analysis set; IMP = investigational medicinal product; PPS = per protocol set; SAF = safety population

Forty-three subjects were screened for study participation, of whom 25 were considered eligible. One subject (4.0%) was discontinued due to prohibited vitamin B6 intake. Twenty-four subjects (96.0%) completed the study (Fig. 1). Demographics and baseline characteristics of the study population are presented in Table 1. The study population had a mean age of 15.5 years and 14 subjects (56.0%) were female. One subject (4%) was Black or African American and 24 subjects were not (96%). Twenty-four subjects (96.0%) had confirmed PH type 1 due to *AGXT* gene mutations which were categorized as complex heterozygous in nine subjects (36.0%), homozygous in 13 subjects (52.0%) and unknown category in two subjects (8.0%). One subject (4.0%), randomized to placebo, had PH type 2; no subjects with PH type 3 were enrolled.

**Table 1:** Baseline characteristics of study population

	Oxabact N** = **13	Placebo N** = **12	Total N** = **25
**Age (years)**			
Mean (SD)	12.9 (6.4)	18.3 (16.5)	15.5 (12.4)
Min, Max	5, 24	5, 54	5, 54
**Height (cm)**			
Mean (SD)	146.18 (22.38)	148.09 (23.80)	147.10 (22.60)
Min, Max	113.0, 179.0	112.0, 182.0	112.0, 182.0
**Weight (kg)**			
Mean (SD)	45.98 (25.28)	48.36 (23.24)	47.12 (23.84)
Min, Max	18.0, 97.4	20.7, 92.6	18.0, 97.4
**Sex (n[%])**			
Female	9 (69.2%)	5 (41.7%)	14 (56.0%)
Male	4 (30.8%)	7 (58.3%)	11 (44.0%)
**Race (n[%])**			
Black or African American	1 (7.7%)	0	1 (4.0%)
Not Black or African American	12 (92.3%)	12 (100%)	24 (96.0%)
**PH type (n[%])**			
Type 1	13 (100.0%)	11 (91.7%)	24 (96.0%)
Type 2		1 (8.3%)	1 (4.0%)
Type 3	0	0	0
**Time since diagnosis (months)**			
Mean (SD)	101 (75.1)	54.1 (38.1)	78.2 (63.6)
Median (Q1, Q3)	80.3 (37.5, 135)	43.8 (31.5, 73.4)	71.4 (34.7, 110)
**Baseline Pox concentration (µmol/L)**			
Mean (SD)	14.8 (5.7)	14.4 (5.4)	14.6 (5.5)
Median (Q1, Q3)	12.7 (12.3, 15.0)	12.5 (9.8, 20.0)	12.7 (11.0, 17.7)
Min, Max	9, 31	9, 24	9, 31
**eGFR (mL/min/1.73 m**^**2**^**)**			
Mean (SD)	70.3 (11.6)	62.4 (16.9)	66.5 (14.6)
Median (Q1, Q3)	70.9 (62.2, 77.7)	65.4 (50.6, 77.1)	70.8 (60.3, 77.7)
Min, Max	44, 90	28, 81	28, 90
**Baseline Uox excretion (mmol/24 h/1.73 m**^**2**^**)**			
Mean (SD)	2.107 (0.929)	1.759 (0.937)	1.940 (0.931)
Median (Q1, Q3)	1.815 (1.659, 2.462)	1.443 (1.195, 2.085)	1.792 (1.243, 2.188)
Min, Max	0.90, 4.53	1.07, 4.45	0.90, 4.53
**No. of kidney stone events in last 3 years (n[%])**			
0 events	4 (30.8%)	4 (33.3%)	8 (32.0%)
1 event	3 (23.1%)	2 (16.7%)	5 (20.0%)
2 events	2 (15.4%)	1 (8.3%)	3 (12.0%)
3 or more events	4 (30.8%)	4 (33.3%)	8 (32.0%)

*eGFR*, estimated glomerular filtration rate; *Max*, maximum; *Min*, minimum; *Pox*, plasma oxalate; *SD*, standard deviation; *Uox*, urinary oxalate

The mean (SD) age at PH diagnosis was 9.2 (12.4) years. Subjects randomized to Oxabact had a lower mean (SD) age at baseline (12.9 [6.4] years) compared with placebo (18.3 [16.5] years).

The mean time since PH diagnosis in all patients was 78.2 months (~ 6.5 years), with Oxabact subjects having a longer mean (SD) time since PH diagnosis (101 [75.1] months) compared with placebo (54.1 [38.1] months).

Sixteen subjects (64.0%) had stage II CKD. Stage III CKD was reported in six subjects (24.0%) and borderline stage I CKD was reported in three subjects (12.0%). The mean (SD) age at CKD diagnosis was 11.5 (12.7) years. Subjects who received Oxabact had a lower mean (SD) age at CKD diagnosis (7.5 [6.7] years) compared with subjects who received placebo (16.4 [16.4] years).

### Plasma oxalate, estimated glomerular filtration rate and adjudicated kidney stone events

**Fig. 2:**
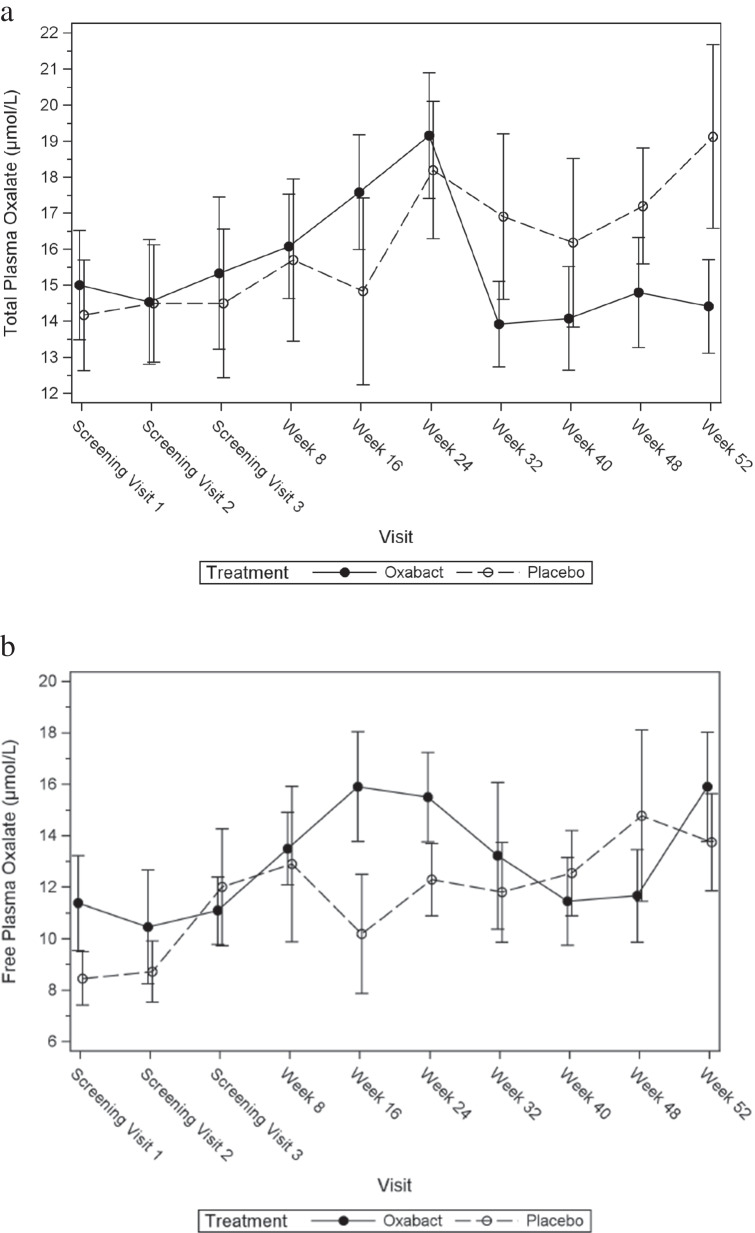
**a** Mean total plasma oxalate over time — full analysis set. **b**. Mean free plasma oxalate over time — full analysis set. Error bars depict standard error

There was no significant difference between the groups in the primary outcome measure, CfB in total Pox concentration at Week 52 (Table 2). The Least Squares (LE) mean estimate (SE) for the CfB in total Pox concentration after 52 weeks was − 3.80 μmol/L (95% CI: − 7.83 to 0.23 μmol/L; *p*-value = 0.064). Although a statistically significant difference in mean total Pox concentrations over time was not observed, total Pox concentrations in the Oxabact group started to decrease after Week 24, yet continued to increase for subjects who received placebo throughout the study (Fig. 2a,b). For the subgroup with eGFR < 60 ml/min/1.73 m^2^ as determined with Cystatin C-based CKid/CKD-EPI 2012, the effect was somewhat more pronounced: Oxabact median CfB µmol/L (Q1, Q3) − 3.3 (− 5.3, − 0.7) vs. placebo 2.7 (− 1.5, 6.5) (see Supplementary Table 5).

**Table 2:** Mixed repeated measures model for change from baseline in total plasma oxalate (µmol/L): least squares means estimates at Week 52

Treatment group	LS mean difference (Oxabact–Placebo)			
	LS Mean (SE)	Estimate (SE)	95% CI	*p*-value
Full analysis set				
Oxabact (n = 12)	–0.788 (1.374)	–3.802 (2.033)	–7.829, 0.225	0.06
Placebo (n = 8)	3.015 (1.613)	-	-	-

*CI*, confidence interval; *LS*, Least Squares; *SE*, mean estimate

B6 treatment was distributed similarly in both subgroups: there were a total of 16 patients taking Vitamin B6 at baseline, 9/13 in the Oxabact arm and 7/12 in the placebo arm i.e. 69% in Oxabact arm vs. 58% in the placebo arm. There were no indications that patients in the Oxabact arm taking Vitamin B6 had a better outcome; patients taking B6 Oxabact CfB median Pox µmol/L (Q1, Q3) 1.7 (− 0.7, 3.7) and placebo 2.8 (1.0, 5.7).

**Fig. 3:**
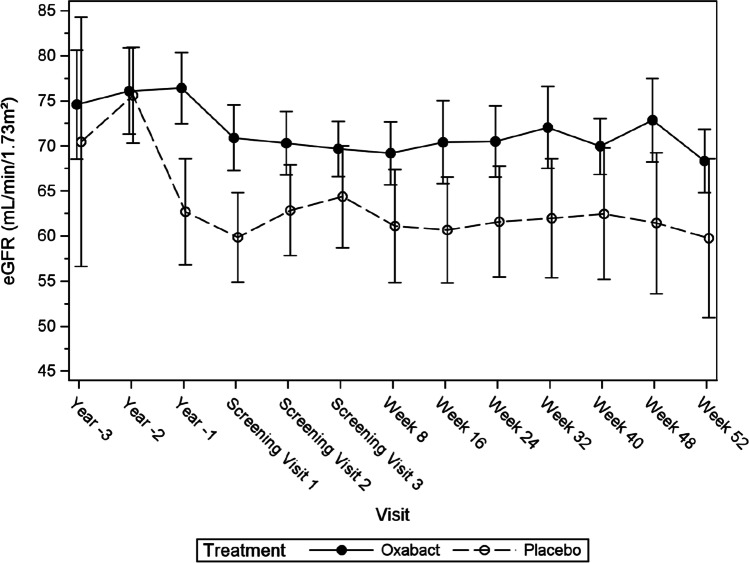
Mean estimated glomerular filtration rate over time — full analysis set, absolute values. Error bars depict standard error. Estimated glomerular filtration rate was calculated using the creatinine-based Bedside Schwartz formula in children (< 18 years) or the Chronic Kidney Disease Epidemiology Collaboration formula for adults (≥ 18 years).

Change from baseline eGFR after 52 weeks of treatment was evaluated as the first key secondary efficacy endpoint. No statistically significant differences were observed between the two treatment arms (*p*-value = 0.744). Estimated GFR was approximately 8 mL/min/1.73 m^2^ higher at baseline in subjects who received Oxabact compared with subjects who received placebo; neither arm showed an improvement or a deterioration over 52 weeks study time (Fig. 3).

The number of kidney stone events occurring in subjects during the 3-year pre-treatment phase is presented in Table 1. There was no statistically significant difference in the number of kidney stone events often experienced by subjects during the study period. However, the incidence rate of adjudicated kidney stone events during treatment was slightly lower in subjects randomized to Oxabact (seven kidney stone events reported for 13 subjects) compared with placebo (eight kidney stone events in 12 subjects; Table 3). The incidence rate difference was − 0.179 (95% CI: − 0.805 to 0.447; *p*-value = 0.12).

**Table 3:** Incidence rate of kidney stone events

Variable	Oxabact N = 13	Placebo N = 12	IRD (95% CI)	IRR (95% CI)
Occurrence	7	8	− 0.179 (− 0.805–0.447	0.749 (0.231–2.362)
Person-time (years)	13.109	11.214	-	-

*CI*, confidence interval; *IRD*, incidence rate difference; *IRR*, incidence rate ratio

### Other study endpoints: urinary oxalate, nephrocalcinosis, cardiac function, O. formigenes quantification, kidney markers and quality of life

**Fig. 4:**
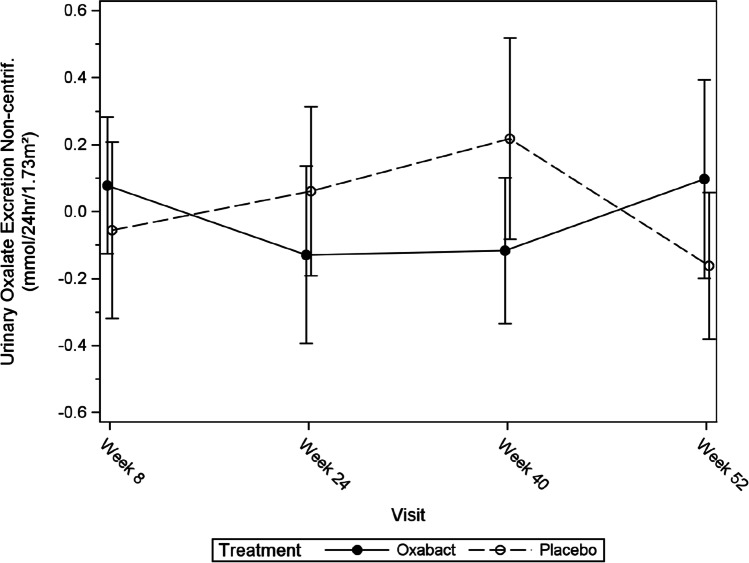
Mean change from baseline of non-centrifuged urinary oxalate excretion over time — full analysis set. Error bars depict standard error

Subjects who received Oxabact had a higher mean (SD) baseline Uox excretion [2.11 (0.93) mmol/24 h/1.73 m^2^] compared with subjects who received placebo [1.76 (0.94) mmol/24 h/1.73 m^2^] (Fig. 4). Over the course of the study, Uox initially decreased in the Oxabact arm and increased in subjects receiving placebo. The trend was reversed after Week 40 and through 52 weeks, when Uox in Oxabact-treated subjects increased to baseline levels, and decreased in placebo subjects (Fig. 3).

There were no marked differences between treatment arms with regard to nephrocalcinosis. Most subjects had no or slight shift in their clinical grading, and there was no change in subjects receiving placebo.

Myocardial function, as measured by both TE and STE, was considered normal in all subjects at baseline. Numeric improvements in LVEF and GLS within the normal range were observed in Oxabact-treated subjects.

Change from baseline in the number of *O. formigenes* bacteria in stool samples was determined by qPCR; imputation was performed due to some data points being below the limit of detection for both genotype 1 and genotype 2. At Week 52, the median cell count for genotype 1 in the Oxabact arm was 0.7740 × 10^6^ cells/g (CfB of 0.69 × 10^6^ cells/g). By contrast, there were 0.0834 × 10^6^ cells/g (CfB of 0.00 × 10^6^ cells/g) in the placebo arm. At Week 52, the median value for genotype 2 was 0.0268 × 10^6^ cells/g (CfB of 0.00 × 10^6^ cells/g) in both treatment arms.

Summary results for urinary and plasma parameters related to kidney function, kidney tubular capacity and inflammation are presented in Supplementary Tables 1 and 2, respectively. Kidney stone–related urinary parameters (excretion of citrate and calcium, urinary volume, osmolality and pH) as well as urinary kidney function-related parameters (excretion of creatinine and glycolate) may indicate a higher excretion of oxalate and a stabilized kidney function in subjects who received Oxabact compared with subjects who received placebo.

A number of plasma kidney biomarkers (calcium, citrate, creatinine and bicarbonate) as well as inflammatory markers (leukocytes) were stabilized in response to Oxabact compared with the placebo arm. These changes may indicate a response effect to Oxabact treatment related to an improvement in kidney function and a reducing inflammatory state (see Supplementary Tables 1 and 2). The small decrease in mean plasma glycolate in the placebo arm may be driven by one low eGFR patient with highly elevated plasma glycolate at baseline (CfB − 292 µmol/L). The urinary glycolate excretion shows a substantial difference between the groups, + 20.3 µmol/24 h/1.73 m^2^ (Oxabact) vs. − 231 µmol/24 h/1.73 m^2^ (placebo). Hydration status improved in subjects who received Oxabact compared with placebo, and kidney function parameters (specifically citrate and creatinine in plasma) showed a smaller increase in the Oxabact arm versus the placebo arm, although no difference in cystatin-C or eGFR was noted.

In the Oxabact arm, mean scores on the physical and mental component of the SF-36v2 started below average on the USA norm-based range (< 50) but rose above average (> 50) from Week 24 onwards. In the placebo arm, mean scores stayed below average (< 50) throughout the study with a CfB at Week 52 of 6.0 and 6.3, respectively. Mean scores in the placebo arm stayed below average (< 50) throughout the study. On the norm-based physical health score scale and the norm-based psychosocial health score scale of the parent-reported CHQ/PF50, mean scores tended to stay below the USA norm-based range (< 50) in both treatment arms.

### Post-hoc analysis

At baseline, median ratio of total:free Pox (SD) was 1.45 (0.48) in subjects who received Oxabact and 1.56 (0.48) in subjects who received placebo, indicating that total Pox was approximately 45–55% higher than free Pox. By Week 52, the median ratio decreased to 1.06 (0.33) in Oxabact-treated subjects and remained similar to baseline values at 1.45 (0.33) in placebo subjects (Supplementary Table 4). This indicates that CaOx crystals and protein-bound oxalate was reduced in the Oxabact arm.

Fractional excretion of oxalate (FEOx = (Urine oxalate concentration × Serum creatinine × 100)/(Total plasma oxalate × Urine creatinine)) was calculated to better understand the development of total Pox and Uox excretion across treatment arms and study duration. The mean (SD) fractional excretion of Uox (FeOx) was similar in both treatment arms at baseline: 121.5 in the Oxabact arm and 119.7 in the placebo arm. At Week 52, FeOx increased in the Oxabact arm (136.4), and decreased in the placebo arm (106.8), indicating a higher excretion of oxalate in the Oxabact arm.

### Safety assessments

An overview of AEs and serious adverse events (SAEs) is presented in Table 4. Fewer AEs and SAEs were reported in the Oxabact arm (46 AEs in 13 subjects; 5 SAEs in three subjects) than in the placebo arm (61 AEs in 11 subjects; 10 SAEs in four subjects). Most AEs were considered to be unrelated to the study treatment and Grade 1 (mild) to Grade 2 (moderate) in intensity. Two AEs were considered at least possibly related to study treatment in the Oxabact arm and 3 in the placebo arm. More kidney and urinary AEs of higher severity (Grade 2 or 3) were noted in the placebo arm compared with Oxabact (18 vs. 9 Grade 2 AEs, and 10 vs. 6 Grade 3 AEs in placebo vs. Oxabact, respectively). There were no related SAEs, fatal AEs nor AEs leading to discontinuation of study drug/study in any of the treatment arms.

**Table 4:** Adverse and serious adverse events

System organ class preferred term	Oxabact N = 13		Placebo N = 12	
	**n (%)**	**m**	**n (%)**	**m**
Adverse events				
Renal and urinary disorders	4 (30.8%)	10	4 (33.3%)	9
Calculus urinary	1 (7.7%)	1	2 (16.7%)	2
Nephrolithiasis	1 (7.7%)	1	2 (16.7%)	2
Renal colic	2 (15.4%)	7	1 (8.3%)	2
Renal impairment	-	-	1 (8.3%)	2
Ureterolithiasis	1 (7.7%)	1	-	-
Urinary tract obstruction	-	-	1 (8.3%)	1
Any serious adverse event	3 (23.1%)	5	4 (33.3%)	10
Renal and urinary disorders	2 (15.4%)	3	4 (33.3%)	8
Calculus urinary	-	-	2 (16.7%)	2
Renal colic	1 (7.7%)	-	1 (8.3%)	2
Nephrolithiasis	-	-	1 (8.3%)	1
Renal impairment	-	-	1 (8.3%)	2
Ureterolithiasis	1 (7.7%)	-	-	-
Urinary tract obstruction	-	-	1 (8.3%)	1
Infections and infestations	2 (15.4%)	-	1 (8.3%)	1
Bacterial pyelonephritis	-	-	1 (8.3%)	1
Escherichia urinary tract infection	1 (7.7%)	1	-	-
Pyelonephritis acute	1 (7.7%)	1	-	-
Musculoskeletal and connective tissue disorders	-	-	1 (8.3%)	1
Flank pain	-	-	1 (8.3%)	1

*m*, number of events; *n*, number of subjects

## Discussion

Poor clinical prognosis for patients suffering with PH necessitates an effective therapeutic intervention that prevents or delays the formation and deposition of CaOx crystals in affected organs. Previous studies with Oxabact [26, 27] revealed unexpected effects of OC5 including a consistent increase in urinary excretion of oxalate and calcium vs. placebo. Post-hoc analyses further revealed increasing total Pox in the placebo group, while Pox concentrations remained stable or decreased in Oxabact-treated subjects (treatment difference between arms: − 3.3 µmol/L, *p*-value = 0.045). Taking into account previously published work on kidney stones [28], a dissolution effect of Oxabact on CaOx crystals was proposed and considered possible based on its hypothesized propensity to reduce the systemic oxalate burden, by enhancing its removal from the plasma via the GI tract. Therefore, the present study was conducted over a 12-month period to attempt to confirm a dissolution effect on CaOx precipitates. Considering the slow progression rate and related slow increase of Pox in most patients, a population with established CKD (eGFR < 90 mL/min/1.73 m^2^) was chosen for this study.

This randomized controlled study investigated the efficacy of Oxabact in reducing total Pox in patients with a diagnosis of PH over 12 months of treatment, but it did not reach the primary endpoint (*p*-value = 0.064, treatment difference − 3.8 µmol/L). As in the previous studies, plasma oxalate seemed to increase over time in the placebo arm but seemed to remain stable in the Oxabact arm (*p* = 0.064). Consistent with earlier studies [26, 27], total Pox levels seemed stable in the Oxabact arm as hypothesized upon its proposed mechanism of action. Careful and timely treatment of samples with HCl ensured that the measured Pox levels were related to physiological activity and did not arise as an artifact of inadequate sample handling and storage [29]. For the subgroup with eGFR < 60 ml/min/1.73 m^2^ as determined with Cystatin C-based CKid/CKD-EPI 2012, the effect was somewhat more pronounced: Oxabact median CfB µmol/L (Q1, Q3) − 3.3 (− 5.3, − 0.7) vs. placebo 2.7 (− 1.5, 6.5). This indicates that patients with better eGFR may have influenced the variation in plasma oxalate levels from baseline, as GFR is a major factor, and potentially could even have masked Oxabact action in this study (see Supplementary Table 5).

*O. formigenes* metabolizes oxalate but does not affect its synthesis and therefore, a relative decrease in total Pox concentration is unlikely to be detected until oxalate deposits are substantially depleted [26]. An initial rise in Pox observed in both groups may be due to the mobilization of stored oxalate before a reduction in levels in response to Oxabact treatment was observed. This was further confirmed post-hoc by a decreasing ratio of total Pox:free Pox in the Oxabact arm, suggesting a depletion of crystals or protein bound oxalate in the Oxabact arm after 12 months.

Over the course of the study, we observed small changes in Uox excretion. With Oxabact treatment, we saw an initial decrease in Uox excretion, followed by a small increase from week 40–52. The inverse was true for the placebo arm. While this could be a result of the naturally high variability in Uox [30], all three previous placebo-controlled Oxabact studies over 6–12 months have reported the treatment group to show slightly higher Uox excretion vs. placebo at the end of the study [19, 26, 27]. Some patients improved eGFR during the first 8 weeks of the study (10 patients had a median increase in eGFR (Q1, Q3) of 5.0 (4.5, 10.0) ml/min/1.73 m^2^), probably a study effect from better compliance with fluid intake, which also is seen in the urinary osmolality (see Supplementary Table 1). A recent rat study with *O. formigenes* found a similar effect [31]. Therefore, this finding is consistent with earlier work on Oxabact.

Contrary to results from previous studies, no difference in kidney function (eGFR) was observed between treatment arms at the end of the study. It was reported that hydration status improved during the study (see Supplementary Tables 1 and 2), possibly explaining the large variability in eGFR values observed. In a 5-year-old subject who received placebo, eGFR increased by 40 mL/min/1.73 m^2^ during the baseline period, and another 20 mL/min/1.73 m^2^ during the treatment period. Considering that the proposed eGFR treatment effect for this study had been estimated to be between 2 and 5 mL/min/1.73 m^2^, the issue of stable hydration causes practical hurdles for accurate eGFR determination in a small study population. Future studies should control or compensate for hydration status and possibly accommodate a longer treatment duration in order to determine the treatment effects on eGFR accurately in PH populations.

Despite no observed difference in eGFR between the two treatment arms, subjects receiving Oxabact but not placebo showed levels of biomarkers indicative of stable kidney function. Oxabact patients had higher Uox and slightly higher kidney stone burden in the years before their participation in this study, which is also supported by a younger age of diagnosis, but had fewer symptomatic stone events during the treatment period when compared with placebo, in line with previous studies [27]. The patients assigned to Oxabact were generally more severely affected, and the combination of safety data, urinary and kidney parameters and stone burden indicate a potential clinical benefit.

The effect of Oxabact on nephrocalcinosis and cardiac function was assessed by ultrasound. No difference in nephrocalcinosis grading was observed between the two treatment arms, which suggests that Oxabact is unable to improve nephrocalcinosis over 52 weeks in a population with established CKD. Evidence is lacking within the literature as to whether established nephrocalcinosis is reversible and therefore an absence of improvement in nephrocalcinosis is not unexpected. Assessment of echocardiography parameters indicated that both LVEF and GLS values improved slightly in the Oxabact arm during the study, whereas values worsened slightly in the placebo arm. This finding echoes the previous Phase II study results in which an improvement and stabilization of cardiac function was observed in subjects treated with Oxabact [12].

Other parameters pointed toward a small treatment benefit of Oxabact. Markers for kidney function, kidney tubular capacity and inflammation in urine and plasma generally indicated a CfB stabilization in the Oxabact arm compared with placebo. Supplementary Table 1 shows that substantial differences were found for urinary citrate, urinary osmolality and for urinary calcium. According to Zhao et al., 2016 [23], PH-patients have lower urinary calcium and higher urinary volume with lower osmolality compared to the reference population, which could be explained by calcium binding to oxalate along with progressive failure of the kidney to concentrate urine. Differences versus placebo support the theory that Oxalobacter may be dissolving renal CaOx and increasing Ox^2−^ (and Ca^2+^) in urine. The beneficial effect of Oxabact was further supported by fewer SAEs and fewer kidney/urinary tract treatment–emergent AEs in the Oxabact arm versus the placebo arm.

Therefore, while Oxabact appeared in the early phase of treatment to afford moderate benefit, over a longer timeframe, a more sustained effect could potentially be achieved. If so, there may be benefit from Oxabact therapy in PH1 patients who have already received liver transplantation, or possibly also in patients with enteric hyperoxaluria.

The main limitations to the study were the small sample size, distribution in different hospitals, study duration and the highly heterogeneous disease progression rates among individuals which may have impacted clinical outcome in response to treatment [23], as well as patient dropout mainly from the placebo arm at the end of the observation period. A major study bias was the presence of kidney stone episodes, surgical procedures or interventions (J-catheters), pyelonephritis or UTIs, and transient eGFR worsening that may impact on plasma oxalate levels whenever that happened close to a study visit. A high incidence of comorbidities (e.g., kidney stones, urinary tract infections) in patients with PH often necessitates treatment interventions requiring hospitalization and antibiotics, limiting Oxalobacter survival and thus Oxabact efficacy. Several of the patients in this trial continued into a 2-year open-label follow-up study (OC5-OL02). Although the study terminated prematurely, available data showed a sharp rise in Pox levels (> 20 µmol/L) in four subjects treated with Oxabact for 12–24 months (data not shown). Two of these subjects, in whom eGFR was also affected, had been hospitalized repeatedly during the study and treated with cephalosporin, fosfomycin or other antibiotics to which *O. formigenes* is sensitive shortly before some study visits, which could impact on Oxabact efficacy. Poor adherence to the study protocol and the treatment of urinary tract infections could have resulted in the use of antibiotics that compromised the gut microflora. In addition, although PCR analysis confirmed resident *O. formigenes* gut colonization within the Oxabact treatment group, this method does not indicate the active metabolism of oxalate by the bacteria. Future studies may want to consider alternative assays capable of detecting live cells. Finally, despite efforts to include subjects with each of the three PH types, only subjects with PH type 1 were treated with *Oxalobacter formigenes *(Oxabact^TM^). A greater treatment effect may have been observed in subjects with PH types 2 and 3 due to them having slower progression of endogenous deposition, or in enteric hyperoxaluria.

Future studies may use different clinical endpoints. As previously mentioned, using eGFR as an endpoint can cause concerns due to dehydration in study subjects, and the effect of eGFR on plasma oxalate. Additionally, an endpoint relating to kidney stone events may be problematic due to the potential dissolution of crystals that could decrease the size of a stone and cause the stone to pass; a beneficial event could therefore be reported as an adverse finding. The authors are proposing that the recommended clinical outcome endpoint in PH populations should be the number and size of kidney stones, not events, which would require a larger sample size and treatment in an open-label study for at least 4 years. A longer study extension presumably will allow better evaluation of Oxabact benefit in the long term.

Finally, the observation of plasma oxalate decreases in patients with mild CKD using an oral and well-tolerated drug could justify a new trial to evidence the potential benefit of very early and sustained treatment in preventing or decreasing stone formation, and subsequent infection, inflammation and obstruction, preserving kidney function in the long-term.

In conclusion, although this study failed to provide evidence supporting the overall efficacy of Oxabact in subjects with PH and maintained kidney function below the lower limit of the normal range, a subtle treatment effect suggests a potential role in preventing the formation of kidney stones within a PH population with maintained kidney function. The delivery of *O. formigenes* as Oxabact or in other formulations therefore has the potential to be used for PH1 patients who have already received liver transplantation, in patients with enteric hyperoxaluria, or, as an adjuvant with other therapies in the treatment of PH.

## Supplementary Information

Below is the link to the electronic supplementary material.

Graphical Abstract (PPTX 170 KB)

Supplementary file2 (DOCX 59 KB)

## Data Availability

The authors confirm that the data supporting the findings of this study are available within the article.
