# Supplementary material for: ePHex: a phase 3, double-blind, placebo-controlled, randomized study to evaluate long-term efficacy and safety of Oxalobacter formigenes in patients with primary hyperoxaluria
Source: Pediatr Nephrol. 2022 May 12;38(2):403–15. doi: 10.1007/s00467-022-05591-5 (PMC9763141; doi:10.1007/s00467-022-05591-5)
Supplement: Supplementary file 1 — Graphical Abstract (PPTX 170 KB) [file 467_2022_5591_MOESM1_ESM.pptx]

## Slide 1
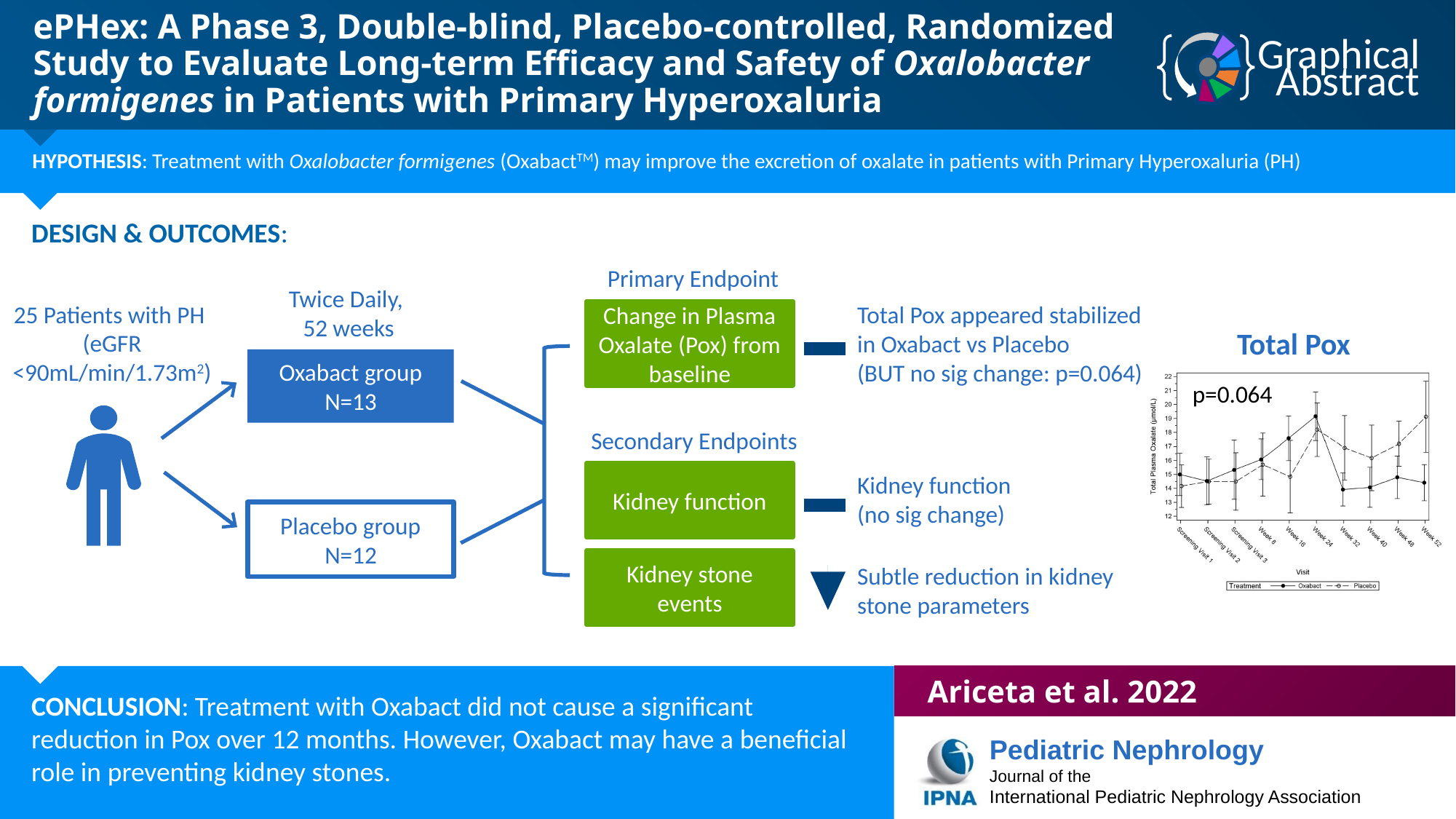

ePHex: A Phase 3, Double-blind, Placebo-controlled, Randomized Study to Evaluate Long-term Efficacy and Safety of Oxalobacter formigenes in Patients with Primary Hyperoxaluria
HYPOTHESIS: Treatment with Oxalobacter formigenes (OxabactTM) may improve the excretion of oxalate in patients with Primary Hyperoxaluria (PH)
DESIGN & OUTCOMES:
Primary Endpoint
Twice Daily,
52 weeks
25 Patients with PH
(eGFR <90mL/min/1.73m2)
Total Pox appeared stabilized in Oxabact vs Placebo
(BUT no sig change: p=0.064)
Change in Plasma Oxalate (Pox) from baseline
Total Pox
Oxabact group
N=13
p=0.064
Secondary Endpoints
Kidney function
Kidney function (no sig change)
Placebo group
N=12
Kidney stone events
Subtle reduction in kidney stone parameters
Ariceta et al. 2022
CONCLUSION: Treatment with Oxabact did not cause a significant reduction in Pox over 12 months. However, Oxabact may have a beneficial role in preventing kidney stones.
