# Supplementary material for: ePHex: a phase 3, double-blind, placebo-controlled, randomized study to evaluate long-term efficacy and safety of Oxalobacter formigenes in patients with primary hyperoxaluria
Source: Pediatr Nephrol. 2022 May 12;38(2):403–15. doi: 10.1007/s00467-022-05591-5 (PMC9763141; doi:10.1007/s00467-022-05591-5)
Supplement: Supplementary file 2 — Supplementary file2 (DOCX 59 KB) [file 467_2022_5591_MOESM2_ESM.docx]

SUPPLEMENTARY INFORMATION

For submission: ePHex: A Phase 3, Double-blind, Placebo-controlled, Randomized Study to Evaluate Long‑term Efficacy and Safety of *Oxalobacter formigenes* in Patients with Primary Hyperoxaluria

In consideration for: *Pediatric Nephrology*

Gema Ariceta, Laure Collard, Saoussen Abroug, Shabbir H. Moochhala, Edward Gould, Abir Boussetta, Mohamed Ben Hmida, Sudarsana De, Tracy E. Hunley, Faical Jarraya, Gloria Fraga, Ana Banos M.Sc., Elisabeth Lindner M.Sc. MBA, Bastian Dehmel M.D., Gesa Schalk

Corresponding author: Dr Gema Ariceta, Hospital Vall d’Hebron, Barcelona, Spain, gariceta@vhebron.net

Supplementary Table 1: Urinary Markers for Kidney Function, Kidney Tubular Capacity and Inflammation, Median Change from Baseline at Week 52 – Full Analysis Set

|  | **Oxabact**  **N=13** | **Placebo**  **N=12** |
| --- | --- | --- |
| **Kidney Stone Parameters^a^** |  |  |
| Urinary calcium (non-centrifuged) (µmol/24 hr/1.73 m^2^) | –120 | –320 |
| Urinary oxalate (mmol/24 hr/1.73 m^2^) | –0.12 | –0.30 |
| Urinary glycolate (µmol/24 hr/1.73 m^2^) | 20.30 | –230.65 |
| Urinary phosphorus excretion (mmol/24 hr/1.73 m^2^) | 2.76 | 1.56 |
| Urinary magnesium excretion (mmol/24 hr/1.73 m^2^) | –0.31 | –0.26 |
| Urinary citrate excretion (µmol/24 hr/1.73 m^2^) | 20 | –110 |
| **Hydration Status** |  |  |
| Urinary volume (mL) | 20 | –180 |
| Urinary Osmolality (mOsm/kg) | –2.75 | –34.00 |
| Urinary urea (µmol/24 hr/1.73 m^2^) | –398 | –206 |
| **Kidney Function** |  |  |
| Urinary creatinine (µmol/24 hr/1.73 m^2^) | 369 | –773 |
| Urinary pH | 0.25 | 0.00 |
| Urinary CaOx crystal excretion (µmol/24 hr/1.73 m^2^) | 0.01 | –0.01 |
| 1. Kidney stone parameters are presented as body surface area‑corrected excretion values | | |

Supplementary Table 2: Plasma Markers for Kidney Function, Kidney Tubular Capacity and Inflammation, Median Change from Baseline at Week 52 – Full Analysis Set

|  | **Oxabact**  **N=13** | **Placebo**  **N=12** |
| --- | --- | --- |
| **Kidney Stone Predictors** |  |  |
| Calcium in plasma (µmol/L) | –50 | 20 |
| Oxalate in plasma (µmol/L) | –0.2 | 2.8 |
| Glycolate in plasma (µmol/L) | 6.0 | –5.0 |
| **Inflammation** |  |  |
| Leukocytes (10^9^/L) | –0.85 | 0.05 |
| CRP (mg/L) | –0.050 | 0.100 |
| **Citrate in Plasma (µmol/L)** | –1.5 | 24.5 |
| **Hydration Status** |  |  |
| BUN (mmol/L) | –0.8 | –0.9 |
| **Kidney Function** |  |  |
| Serum creatinine (µmol/L) | 1.700 | 3.300 |
| Cystatin C (mg/L) | –0.080 | –0.080 |
| Bicarbonate (mmol/L) | –1.5 | 0.5 |
| BUN=blood urea nitrogen; CRP=C-reactive protein. | | |

Supplementary Table 3: Quality of Life SF-36v2 (Subjects ≥18 years) and CHQ/PF50 (Subjects <18 years) – Full Analysis Set

|  | **SF-36v2** | | | | | |
| --- | --- | --- | --- | --- | --- | --- |
|  | **Norm-based Physical Component Summary** | | | **Norm-based Mental Component Summary** | | |
| **Visit** | **Oxabact N=13** | **Placebo N=12** | **Total N=25** | **Oxabact N=13** | **Placebo N=12** | **Total N=25** |
| Baseline (n) | 3 | 4 | 7 | 3 | 4 | 7 |
| Mean (SD) | 47.3 (8.2) | 46.9 (12.2) | 47.1 (9.9) | 45.0 (21.6) | 32.3 (18.5) | 37.8 (19.3) |
| Week 8 (n) | 3 | 2 | 5 | 3 | 2 | 5 |
| Mean (SD) | 48.5 (5.4) | 39.3 (11.4) | 44.8 (8.5) | 44.9 (23.0) | 15.4 (0.9) | 33.1 (22.9) |
| Week 24 (n) | 2 | 2 | 4 | 2 | 2 | 4 |
| Mean (SD) | 52.4 (2.7) | 53.6 (5.2) | 53.0 (3.4) | 52.1 (7.6) | 33.8 (28.5) | 43.0 (20.0) |
| Week 40 (n) | 3 | 3 | 6 | 3 | 3 | 6 |
| Mean (SD) | 54.5 (1.5) | 47.1 (10.4) | 50.8 (7.8) | 53.6 (4.8) | 33.5 (21.5) | 43.5 (17.8) |
| Week 52 (n) | 3 | 2 | 5 | 3 | 2 | 5 |
| Mean (SD) | 53.3 (6.6) | 47.3 (16.5) | 50.9 (10.1) | 51.3 (8.5) | 36.6 (26.4) | 45.4 (16.6) |
|  | **CHQ/PF50** | | | | | |
|  | **Norm-based Physical Health Score** | | | **Norm-based Psychosocial Health Score** | | |
|  | **Oxabact N=13** | **Placebo N=12** | **Total N=25** | **Oxabact N=13** | **Placebo N=12** | **Total N=25** |
| Baseline (n) | 9 | 7 | 16 | 9 | 7 | 16 |
| Mean (SD) | 44.6 (9.7) | 49.2 (4.2) | 46.6 (7.9) | 45.7 (9.3) | 51.1 (9.1) | 48.1 (9.3) |
| Week 8 (n) | 7 | 7 | 14 | 7 | 7 | 14 |
| Mean (SD) | 52.7 (2.2) | 46.8 (4.7) | 49.7 (4.7) | 50.6 (4.0) | 48.4 (10.9) | 49.5 (8.0) |
| Week 24 (n) | 9 | 8 | 17 | 9 | 8 | 17 |
| Mean (SD) | 46.5 (8.6) | 46.1 (8.8) | 46.3 (8.4) | 48.7 (7.5) | 44.4 (9.9) | 46.7 (8.7) |
| Week 40 (n) | 8 | 5 | 13 | 8 | 5 | 13 |
| Mean (SD) | 41.8 (13.0) | 45.8 (3.2) | 43.3 (10.3) | 48.0 (11.0) | 44.7 (17.1) | 46.7 (13.1) |
| Week 52 (n) | 10 | 7 | 17 | 10 | 7 | 17 |
| Mean (SD) | 42.7 (14.7) | 48.9 (4.9) | 45.3 (11.8) | 47.8 (11.0) | 49.7 (13.1) | 48.6 (11.5) |
| CHQ/PF50=child health questionnaire parent form 50 questions; SD=standard deviation; SF-36v2=36-item short form survey.  Note: Baseline was defined as the last scheduled value prior to first dose of study treatment. | | | | | | |

Supplementary Table 4: Ratio of Plasma Oxalate (µmol/L) and Free Plasma Oxalate (µmol/L) over Time – Full Analysis Set

|  |  | **Oxabact (N=13)** | | **Placebo (N=12)** | | **Total (N=25)** | |
| --- | --- | --- | --- | --- | --- | --- | --- |
| **Visit** | | **Observed Value** | **Change from baseline** | **Observed Value** | **Change from baseline** | **Observed Value** | **Change from baseline** |
| Screening (n) | | 13 | - | 11 | - | 24 | - |
| Mean (SD) | | 1.52 (0.54) | - | 1.86 (0.83) | - | 1.67 (0.69) | - |
| Screening Visit 2 (n) | | 13 | - | 11 | - | 24 | - |
| Mean (SD) | | 1.99 (1.64) | - | 1.74 (0.61) | - | 1.88 (1.26) | - |
| Screening Visit 3 (n) | | 10 | - | 12 | - | 22 | - |
| Mean (SD) | | 1.53 (0.74) | - | 1.30 (0.37) | - | 1.40 (0.56) | - |
| Baseline (n) | | 13 | - | 12 | - | 25 | - |
| Mean (SD) | | 1.45 (0.48) | - | 1.56 (0.48) | - | 1.50 (0.48) | - |
| Week 8 (n) | | 12 | 12 | 10 | 10 | 22 | 22 |
| Mean (SD) | | 1.25 (0.46) | –0.24 (0.71) | 1.54 (0.79) | 0.04 (0.55) | 1.38 (0.63) | –0.12 (0.64) |
| Week 16 (n) | | 11 | 11 | 11 | 11 | 22 | 22 |
| Mean (SD) | | 1.23 (0.38) | –0.27 (0.35) | 1.90 (0.92) | 0.39 (1.08) | 1.56 (0.77) | 0.06 (0.85) |
| Week 24 (n) | | 12 | 12 | 10 | 10 | 22 | 22 |
| Mean (SD) | | 1.31 (0.24) | –0.11 (0.35) | 1.51 (0.26) | –0.09 (0.55) | 1.40 (0.26) | –0.10 (0.44) |
| Week 32 (n) | | 9 | 9 | 10 | 10 | 19 | 19 |
| Mean (SD) | | 1.34 (0.46) | 0.05 (0.49) | 1.60 (0.76) | 0.05 (1.07) | 1.48 (0.63) | 0.05 (0.82) |
| Week 40 (n) | | 10 | 10 | 11 | 11 | 21 | 21 |
| Mean (SD) | | 1.27 (0.23) | 0.03 (0.34) | 1.31 (0.40) | –0.25 (0.65) | 1.29 (0.33) | –0.12 (0.53) |
| Week 48 (n) | | 9 | 9 | 9 | 9 | 18 | 18 |
| Mean (SD) | | 1.42 (0.62) | 0.01 (0.49) | 1.47 (0.54) | –0.16 (0.96) | 1.44 (0.56) | –0.07 (0.74) |
| Week 52 (n) | | 10 | 10 | 8 | 8 | 18 | 18 |
| Mean (SD) | | 1.06 (0.35) | –0.19 (0.30) | 1.45 (0.33) | –0.15 (0.60) | 1.23 (0.39) | –0.17 (0.44) |
| SD=standard deviation | | | | | | | |

Supplementary Table 5: Changes in baseline Pox (µmol/L) based on eGFR (ml/min/1.73m^2^) as determined by Schwartz/CKD-EPI 2009 and Cystatin C-based CKid/CKD-EPI 2012- Full analysis set

|  | **Oxabact Pox,**  **Median (Q1, Q3)**  **eGFR**  ***Schwartz/CKD-EPI 2009,*** **µmol/L** | **Placebo Pox,**  **Median (Q1, Q3)**  **eGFR**  ***Schwartz/CKD-EPI 2009, µmol/L*** | **Oxabact Pox,**  **Median (Q1, Q3)**  **eGFR *Cystatin C CKid/CKD-EPI 2012, µmol/L*** | **Placebo Pox,**  **Median (Q1, Q3)**  **eGFR *Cystatin C CKid/CKD-EPI 2012, µmol/L*** |
| --- | --- | --- | --- | --- |
| Baseline, patients eGFR >60 ml/min/1.73 m^2^ | (n=12)  12.5 (11.8, 16.2) | (n=7)  11.0 (9.0, 14.3) | (n=8)  13.3 (10.7, 16.2) | (n=7)  11.0 (9.0, 14.3) |
| **Change from baseline** **at 52 weeks,** patients eGFR >60 ml/min/1.73 m^2^ | (n=11)  **–0.7** (–3.3, 2.0) | (n=4)  **2.8** (1.8, 3.3) | (n=7)  1.7 (–2.3, 3.7) | (n=4)  2.8 (1.8, 3.3) |
| Baseline, patients eGFR <60 ml/min/1.73 m^2^ | (n=1)  15.0 (15.0, 15.0) | (n=5)  20.7 (13.3, 21.3) | (n=5)  12.7 (12.3, 15.0) | (n=5)  20.7 (13.3, 21.3) |
| **Change from baseline at 52 weeks**, patients eGFR <60 ml/min/1.73 m^2^ | (n=1)  **6.0** (6.0, 6.0) | (n=4)  **2.7** (–1.5, 6.5) | (n=5)  **–3.3** (–5.3, –0.7) | (n=4)  **2.7** (–1.5, 6.5) |

eGFR=estimated glomerular filtration rate; Pox=plasma oxalate
